# Factors Affecting Cervical Cancer Screening Behaviors Based on the Precaution Adoption Process Model: A Qualitative Study

**DOI:** 10.5539/gjhs.v8n6p211

**Published:** 2015-11-17

**Authors:** Afshin Bahmani, Mohammad Hossein Baghianimoghadam, Behnaz Enjezab, Seyed Saeed Mazloomy Mahmoodabad, Mohsen Askarshahi

**Affiliations:** 1 Department of Health education, Faculty of Health, Shahid Sadoughi University of Medical Sciences, Yazd, Iran; 2 Research Center for Nursing and Midwifery care, Department of Midwifery, Nursing and Midwifery School, Shahid Sadoughi University of Medical Sciences, Yazd, Iran; 3 Department of Statistic and Epidemiology, Shahid Sadoughi University of Medical Sciences, Yazd, Iran

**Keywords:** screening, cervical cancer, precaution adoption process, qualitative study

## Abstract

One of the most preventable cancers in women is cervical cancer. Pap smear test is an effective screening program; however, it is not conducted very frequently. The aim of this study is explaining the determinants affecting women’s participation in the Pap smear test based on precaution adoption process model with a qualitative approach. This study was a qualitative approach using a Directed Content Analysis methodology which was conducted in 2014. Participants were 30 rural women who participated in this study voluntarily in sarvabad, Iran. Purposive sampling was initiated and continued until data saturation. Semi-structured interviews were the primary method of data collection. Data were analyzed using qualitative content analysis and continuous comparisons. Women`s information and awareness about cervical cancer and Pap smear is insufficient and most of them believed that they were not at risk; however, they perceived the severity of the disease. Some of them had no adequate understanding of the test benefits. They pointed to the lack of time, financial difficulties, fear of test result and lack of awareness as the main barriers against the Pap smear test; however, they did not say that they were not willing to do the test. Findings could help health policy makers to find the right area and purpose to facilitate the participation of women in the Pap smear test.

## 1. Introduction

Cervical cancer is the second most common cancer among women in the world. Almost half a million new cases are found annually, killing 274000 people in the world (World Health Organization, 2013). Generally cervical cancer is one of the major public health problems and according to the statistics; it is of the ten most common cancers in the world. Based on the performed classifications, cervical cancer is in the third place in the developed countries and has the second place in developing countries (10th Malaysia Plan, 2010). Eighty-five percent of cervical cancer cases occur in developing countries. The estimated global incidence of cervical cancer is 11.7%., the highest prevalence is in Africa (24%), East Europe (21.4%), and Latin America (16.1%) (Bruni et al., 2010). The incidence of cervical cancer in Iran and other Muslim countries is low (Arbyn et al., 2011); however, the mortality rate in Iran is 44% which is similar to Saudi Arabia (44%) and Libya (66%) (GLOBOCAN, 2008). The highest incidence and mortality of cervical cancer is in developing countries because cancer prevention and control programs in these countries are insufficient. In the developed countries, about 80% of cervical cancers have declined implementing effective prevention programs and effective treatment in the early stages (Hoory et al., 2008). Primary and secondary preventive health behaviors can improve overall health outcomes; primary preventive health behavior in cervical cancer includes physical activity and secondary preventive health behaviors include Pap smear test for quick diagnosis which may markedly reduce the effects of the disease (Naslund et al., 1993). Pap smear test is a screening test for cervical cancer diagnosis in apparently healthy women and is considered as a health behavior and health promotion. Firstly, this test is carried out annually for women who are sexually active for three years, if the condition was desired and then it should be repeated every three years (Alam et al., 2007). By introducing the Pap smear as a screening method for cervical cancer since 1950, the prevalence of this disease has decreased to less than 79% (World Health Organization, 2006). Also, screening in several countries for five years has shown that Pap smear test is able to reduce the mortality rate of cervical cancer to up to 60% (Coughlin et al., 2006). Despite the availability and widespread use of Pap smear test, subgroups of high-risk women (including less educated and low income women) are screened rarely (Cervical Cancer Control, 1991). In the developing countries, compared with 63% in the developed countries, Pap smears coverage is roughly 19% (Gakidou et al., 2008). The purpose of models in health education is to increase awareness, modify people’s attitude and help to change their behavior. In this study, the precaution adoption process model was used as the framework which is a model of health education and health promotion. In turn, it may affect psychological variables such as: awareness, perceived susceptibility, perceived severity, perceived benefits, perceived barriers, perceived self-efficacy., and social norms (Glanz et al., 2008). The PAPM attempts to explain how a person comes to decisions to take action and how he or she translates that decision into action. Adoption of a new precaution or cessation of a risky behavior requires deliberate steps unlikely to occur outside of conscious awareness. The PAPM applies to these types of actions.

Sarvabad is a city in Kurdistan province of Iran bordering Iraq and in terms of Medical facilities it is considered as the most disadvantaged cities in Kurdistan province. Considering the lack of diagnostic medical facilities, lack of education, ignoring the principles of prevention, and sometimes unhealthy lifestyle non-communicable diseases such as cancer has increased in the previous years. After breast cancer, cervical cancer is the second most common cancer among women is in this city. Its incidence rate is estimated at 11.5 % in 100000 in a year. This rate had a threefold increase over the past five years. The coverage of Pap smear is 5.5%., which shows less efficient screening (Unit to prevent and fight against disease, 2014). This study aimed at explaining the determinants affecting women’s participation in Pap smear test based on precaution adoption process model with as a qualitative approach.

## 2. Methods

This study had a qualitative approach using a Directed Content Analysis methodology. Qualitative content analysis is a technique that analyzes the literature systematically. In qualitative content analysis, the purpose is to classify the data obtained from interviews, observational protocols, and taped videos. Content analysis reduces the data and give them structure and order. This is a research methodology that examines words and phrases in the text. Doing content analysis, the researcher identifies the repetition, meaning, and relationships of words and concepts in the text. Then he/she inferences the messages that are included in the text (Hajbagheri et al., 2011).

In this study, precaution adoption process model in behavior of cervical cancer screening is used to extract the codes. Research units were selected purposefully based on Maximum Variability among rural women of Sarvabad. A purposive sampling was initiated and continued up to data saturation. The primary method of data collection in this study was using semi-structured interviews with open questions. Because of flexibility and depth, this kind of interviews is suitable in qualitative research (Haidari et al., 2006). A total of 30 women who passed the inclusion criteria were interviewed. Interviews were carried out between September-October 2014 to February-March 2015. The inclusion criteria were: having 20years of age and being married for at least 3 years or more. Exclusion criteria were: Women who did not have the above criteria and had dementia, amnesia, schizophrenia, depression. Duration of the interviews varied from twenty one minutes to forty minutes. Interviews continued until data saturation. Initially, the interview started with open questions, generally based on the theoretical framework of the model. For example, questions on the familiarity with cervical cancer and its extent. Or “can you tell us what type of test is the Pap smear?” In conducting the interviews, the researchers tried to minimize interference in the interview process. Also to deepen the interview, questions such as, can you explain more? What do you mean? Can you give us an example? Were added to the questions asked.

All interviews were recorded and transcribed verbatim immediately for analysis. Considering that in qualitative research it is needed that the researcher be immersed in information (Hsieh et al., 2005) so the researcher listened to the interviews on several occasions and reviewed their typed text over and over.

In this study, different methods were used to approve the data analysis, such as prolonged engagement, Immersion Research., member check, and the peer debriefing.

At the beginning of the interview participants were informed about the purpose of the research, interviews, ensuring the confidentiality of information, and their right to participate or withdraw from the study. Then informed consent was obtained from the participants. The interview time was adjusted based on interviewer request due to their daily work routines.

This study is part of a doctoral dissertation in health education and promotion in Yazd University of Medical Sciences., which was approved by the ethics committee of the university.

## 3. Results

Basic concepts and codes were extracted after analyzing each interview. After browsing, summering and comparing, these codes were classified according to their relevance and similarity. The codes were placed under subcategories of the main theme of precaution adoption process model including; awareness, perceived susceptibility, perceived severity, perceived benefits., perceived barriers., perceived self-efficacy., and social norms. The individual characteristics of the participants are displayed in Table 1.

**Table 1:** Individual characteristics of women participating in the qualitative study in percent

Insurance coverage	Contraception	The average age	Average number of children	Average BMI
96.66 %	66.6%	38.6± 8.14	2.60± 1.47	28.87± 5.34

**Theme 1: Knowledge and awareness:** Participated women had limited and inadequate awareness about cervical cancer. They were not aware of the causes of the disease, how to prevent them, as well as the Pap smear test. This theme had the following sub-themes.

**Lack of awareness of disease transmission:**

A participant said,” people who washed their underwear not on time contract cervical cancer”. “People who keep animals at home must be more careful” (P2). “Women with circumcised husbands, contract cervical cancer more than others”. “Of course, childbirth can cause the disease” (P15).

**Lack of awareness of disease prevention:**

participant said,” to prevent cervical cancer, after urination I should dry myself immediately with paper towels” (P1). Currently, cancer can not be prevented unless the science progress” (P16). If a person have proper nutrition never have diagnosed with cancer.” (P29).

**Theme 2: perceived susceptibility:** Most women had a lower perception about the risks of getting cervical cancer. This theme had the following sub-themes.

**Wrong Cultural beliefs:**

One participant quoted “I’m strong and did not think about the diseases”, “I feel you will be getting the disease if think about it” (P1). Some of the women believed in destiny and ignored the risk of the disease. A participant said, “I probably do not get the disease unless God wants, because the fate of humans is in the hands of the lord” (P7). I am not infected with cervical cancer because I do not use the public bathroom” (P5).

**Assuming genetic disease:**

Some women believed in genetic factors and believed that other factors are ineffective in pathogenesis. A participant said that “I don’t think I get cancer because I have not hereditary background and people who have hereditary background are more at risk” (P3).

**Theme 3: Perceived severity:** The majority of women believed that cervical cancer was a fatal disease. This theme had the following sub-themes.

**Assuming incurable disease:**

A participant said, “Cancer is a serious disease and It has no survival” (P1). Another participant said, “Cervical cancer is a very dangerous disease, because it cannot be treated” (P3).

**Hysterectomy is synonymous with family breakdown:**

A participant said, “it is a devastating disease because the uterus is removed and humiliation and absurdity remains and she is not a woman any longer” (P15). Another participant said,” the disease is horrific because it disintegrate the family and woman loses the ability to have children, husband, and they cannot do the housework any longer” (P19).

**Theme 4: perceived benefits:** Many of the women perceived the benefits of Pap smear test as a test that can ensure the health of the uterus. This theme had the following sub-themes.

**Rapid diagnosis:**

A participant said, “By doing the test, we will be aware of our physical conditions and in case of any problem, they look for solutions” (P10).

**Sense of health and relaxation:**

A participant said, “The benefits of the Pap smear test is that one knows that she is healthy and it will make a sense of well being and safety” (P2). Another participant said, “By doing the test, we will be aware of our physical conditions and in case of any problem, they look for solutions” (P10). A participant said, “When the test result is good, our morale goes up and we have a better life expectancy” (P18).

**Theme 5: perceived barriers:** this theme had the following sub-themes.

**Lack of knowledge:**

A participant said,“I did not have certain information about this test and not I am not interested to follow it” (P1).

**Fear of a positive test result:**

Another participant said, “I’m fearful, maybe the test is positive and I have cancer” (P6).

**Embarrassment of a physical examination and testing:**

A participant said,” for testing, they may ask me to take off all of my cloths and it makes me uncomfortable” (P8).

**Financial problems:**

Another participant said that, “the cost of testing is very high and I can’t afford it” (P9).

**Theme 6: Desired social norms:** In this part women expressed approval or disapproval of people who were close to them and had an impact on them. This theme had the following sub-themes.

**Positive social support:**

A participant said,” I talked to my mother and she encouraged me to do the test. She said, without the test physicians can not diagnose their patients’ problems” (P7).

**Negative social support:**

Another participant said,” I shared the situation with my husband, but my husband doesn’t want me to do the test. He said that “doing the test was shameful” (P11).

## 4. Discussion

Based on the findings of this study, participants’ awareness about the cervical cancer and Pap smear was inadequate. This finding was consisted with Lee‘s finding about American-Korean women (Lee et al., 2000) and Wong‘s (Wong et al., 2009) findings in Malaysia. However, it is not consistent with the study conducted by Karimi et al (Karimi et al., 2012). Information supporting or giving awareness included a series of information and education to the individual in a particular skill (Al-Arabi, 2006). It made the person to be aware of her self-care, and the prevention and control of the disease (Afkham Ebrahimi et al., 2004). To increase awareness appropriate education programs should be planned.

Most of the participants in this study expressed lower sensitivity to in contracting the disease and did not see themselves at risk. Their reasons were being healthy, having no family history of the disease and their belief in good fate, Bessler et al. (2007) showed a significant association between low levels of perceived susceptibility and low participation of women in the Pap smear test. In their study, women`s reasons for their unwillingness to participate in the Pap smear test were lack of pain and lack of disease symptoms (Bessler et al., 2007). Researchers believed that before encouraging people to take a specific health action, they must know that they are susceptible or under the influence of a risk factor (Karimy et al., 2009). Health educators should explain the risks and negative outcomes so as to establish perceived susceptibility in help-seekers (Karimy et al., 2009).

In the current study, perceived severity in the participants was high and the majority of the participants described cervical cancer as a life threatening disease. This finding was consistent with Abotchie study in Ghanaians women (Abotchie et al., 2009), Burak study in American students (Burak & Meyer, 1997), Barata study in Canada (Barata et al., 2008), and ice vendors in Qazvin. In the Mcfarlands et al study, 57% of the subjects said cervical cancer was incurable and 50% believed that the diagnosing was equivalent to death and 37% believed its diagnosing was equivalent to hysterectomy (McFarland, 2003). To decrease fear and anxiety of participation in the screening program, these erroneous beliefs should be considered in Pap smear counseling programs.

In this study, participants believed that the health of their uterus and rapid diagnosing of the disease was among the perceived benefits of the test. Perceived benefits play an important role in creating behavioral changes, particularly health behaviors (Sullivan et al., 2008). In similar study, 64% of the participants considered timely diagnosis of the disease as a strong point (Karimi et al., 2012).

In this study the barriers for Pap test perceived by the participants were; inadequate education about cervical cancer, financial problems, work engagement, lack of time, fear of a positive test result, and embarrassment in doing a physical examination. The participants expressed embarrassment in doing a physical examination, fear of a positive test result, and financial problems as barriers to do the Pap test which is consistent with the findings of our study (Jouzi et al., 2013). In Maatita study in Jordan (Maaita et al., 2012) and Holroyd in China (Holroyd et al., 2004) and also in Fernandez study., (Fernandez-Esquer et al., 2003) results showed that fear of a positive test result and lack of belief to cancer curability can be a major obstacle in doing a Pap smear test. The findings of the studies conducted in Latin America have shown that the embarrassment of a physical examination was the main obstacle for women’s participation in the Pap smear test (Lewis et al., 2004; Agurto, 2001; Bingham et al., 2003).

Regarding perceived self-efficacy., the majority of the participants in this study believed that the test was easy and they could do the test. Several studies have shown that self-efficacy is one of the most important factors, especially in doing Pap smear, of health behaviors (Floyd et al., 2000; Hodgkins et al., 1998; Herath et al., 2009). One of the ways that we can improve the efficiency of health behavior is to reduce the barriers that exist in the way of behavior (Ackerson et al., 2009). So this point should be considered especially in plans that are designed to improve the Pap smear test.

Regarding women`s perceived social norms., our study showed that most of the comments made by family members., particularly comments made by the partners were important. Actually, family is the first and most important source of support (Zamanzadeh et al., 2007). Spouse`s and close friends ’positive feedback can have a central role on the health and performance of the individuals and is effective in reducing stress and increasing satisfaction in lif (Harden et al., 2002).

One of the limitations in this study includes lack of generalize ability of the results to the entire population and another limitation was the absence of similar studies in the field of quality in Iranian women.

## 5. Conclusion

Findings could help health policy makers to find the right area and purpose to facilitate the participation of women in the Pap smear test. It is recommended, before taking any action, if possible, intervention qualitative study done in that regard to the next steps to be taken according to the needs and characteristics of the target population.
